# Mirror effect of genomic deletions and duplications on cognitive ability across the human cerebral cortex

**DOI:** 10.21203/rs.3.rs-8099338/v1

**Published:** 2025-11-28

**Authors:** Kuldeep Kumar, Sayeh Kazem, Guillaume Huguet, Worrawat Engchuan, Jakub Kopal, Thomas Renne, Omar Shanta, Bhooma Thiruvahindrapuram, Jeffrey MacDonald, Josephine Mollon, Laura M Schultz, Emma E M Knowles, David Porteous, Gail Davies, Paul Redmond, Sarah Harris, Simon Cox, Gunter Schumann, Zdenka Pausova, Celia Greenwood, Tomas Paus, Stephen Scherer, Laura Almasy, Jonathan Sebat, David Glahn, Guillaume Dumas, Sebastien Jacquemont

**Affiliations:** Centre de recherche CHU Sainte-Justine and University of Montréal, Canada; Univeristy of Montreal; Centre Hospitalier Universitaire Sainte-Justine Research Center; The Centre for Applied Genomics, The Hospital for Sick Children, Toronto, Ontario, Canada; University of Oslo; University of Montreal; University of California San Diego; Hospital for Sick Children; Boston Children’s Hospital; The Children’s Hospital of Philadelphia; Boston Children’s Hospital; University of Edinburgh; The University of Edinburgh; Lothian Birth Cohorts, Dept. Psychology; Lothian Birth Cohorts, Dept. Psychology; King’s College London; Centre de recherche CHU Sainte-Justine, and Department of Pediatrics, University of Montréal, Canada; Department of Human Genetics, McGill University, Montreal, Quebec; University of Montreal; Hospital for Sick Children; Children’s Hospital of Philadelphia; University of California, San Diego; Department of Psychiatry, Boston Children’s Hospital and Harvard Medical School, Boston, MA 02115;Harvard Medical School, Boston, MA 02115; Centre de recherche CHU Sainte-Justine and University of Montreal, and Mila, Quebec Artificial Intelligence Institute; Université de Montréal

## Abstract

Cognitive deficits are common across many neurodevelopmental and psychiatric conditions, including those studied in the current set of PGC-CNV papers. How changes in regional gene expression across the cerebral cortex influence cognitive ability remains unknown. Population variation in gene dosage—which significantly impacts gene expression—represents a unique paradigm to address this question. We developed a cerebral-cortex gene-set burden analysis (CC-GSBA) to associate a trait with genomic deletions and duplications that disrupt genes with similar expression profiles across 180 cortical regions. We performed CC-GSBA across 180 cortical regions to test associations with cognitive ability in 260,000 individuals from general population cohorts. Most cortical gene sets were associated with a decrease in cognitive ability when deleted or duplicated, and this novel approach revealed opposing cortical patterns for the effect sizes of deletions and duplications. These cortical patterns of effect sizes followed the cortical gradient previously characterized at the molecular, cellular, and functional levels. We show that genes with preferential expression in sensorimotor regions demonstrated the largest effect on cognition when deleted. At the opposing end of the cortical gradient, genes with preferential expression in multimodal association regions affected cognition the most when duplicated. These two gene dosage cortical patterns could not be explained by particular cell types, developmental epochs, or genetic constraints, highlighting the fact that the macroscopic network organization of the cerebral cortex is key to understanding the effects of gene dosage on cognitive traits.

## Background

Neurodevelopmental and psychiatric conditions, including those studied in the current set of PGC-CNV papers^1,2^, are frequently associated with cognitive deficits^3^. Cognitive ability arises from large-scale brain networks^4–7^, which integrate a diverse range of microscale functions influenced by genetic variations. Recent studies have linked spatial patterns of gene expression in healthy individuals^8^ to structural and functional macroscale brain networks. However, despite significant progress, the role of variations in gene expression across the cerebral cortex in underpinning cognitive ability remains unknown. Specifically, in the absence of large transcriptomic datasets of brain tissue with corresponding phenotypes, a direct association between variation in transcription levels and cognition remains out of reach.

To circumvent this issue, we take advantage of two opportunities. First, over 10%^9,10^ of the general population carries a genomic deletion or duplication fully encompassing one or more coding genes. These deletions and duplications are associated with large decreases and increases (> 1.5 to 2.5 standard deviations)^11^, respectively, of the expression of genes they encompass^12^. Second, functional genomic resources such as the Allen Human Brain Atlas (AHBA), BrainSpan^13^, and Human Protein Atlas^14,15^ (HPA) provide information on the spatial distribution of gene expression, allowing us to identify brain regions where genes are preferentially expressed.

Previous studies have estimated that a large proportion of the coding genome is associated with lower cognitive abilities when deleted or duplicated^9,10,16,17^. Most of our current knowledge of gene functions involved in cognitive abilities stems from studies investigating ontologies at the cellular, subcellular, and molecular level, i.e., genes associated with intellectual disability are enriched in chromatin remodelling, gene regulation, and neuronal communication processes^18,19^. We postulate that information on spatial profiles of gene expression across the human cerebral cortex will provide novel insights into the effect sizes on cognitive ability of genomic deletions and duplications. To test this hypothesis, we developed a cerebral-cortex gene-set burden analysis (CC-GSBA) to associate cognitive ability with genomic deletions and duplications that disrupt genes with similar expression profiles across 180 cortical regions. We implemented CC-GSBA in 260,000 individuals from 6 general population cohorts with measures of cognitive ability. Results demonstrated that most cortical gene-sets were associated with cognitive ability. Deletions and duplication effect sizes showed opposing regional patterns along the cortical gradient, which could not be explained by particular cell types, developmental epochs, or genetic constraint.

## Gene dosage effects on cognitive ability across the cerebral cortex

**Figure 1:**
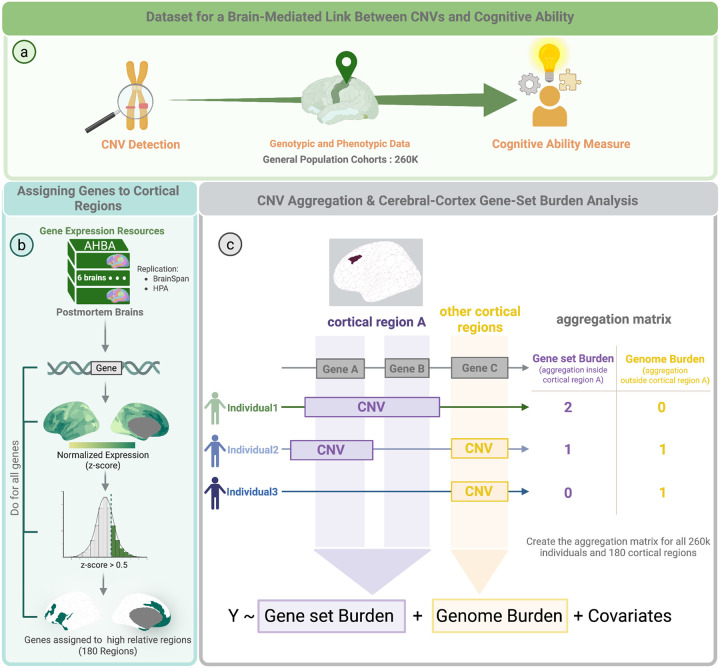
Investigation of the impact of gene expression variation on cognitive ability using Cerebral Cortical Gene Set Burden Analysis (CC-GSBA) **a)** Outline of our methodology for associating CNVs with cognitive ability, leveraging information on where genes are expressed in cortical regions. The analysis integrates genotypic and phenotypic data from six general population cohorts and gene expression data from an independent post-mortem resource, Allen Human Brain Atlas^8^ (AHBA). **b)** Using gene expression data from the AHBA, each gene is initially mapped to cortical regions based on its expression profile and subsequently assigned to regions with preferential expression (Z-score > 0.5). For example, the SST gene shows high expression (Z-score > 0.5) across multiple cortical regions. This process is repeated for all genes (13,678) we have and resulting in 180 gene sets. Replication was assessed using two independent gene expression resources: BrainSpan adult^13^ and Human Protein Atlas^14^ (HPA). c) Then, for each individual in the cohort, CNVs are aggregated both inside and outside the regional gene sets. This allows for the calculation of two distinct burden metrics for each cortical region: Gene-Set Burden (the count of CNVs that overlap with the genes assigned to a specific region) and Genome Burden (the count of CNVs that fall outside the genes of the region of interest, serving as a genome-wide control). Finally, a regression model is applied to test for a region-specific association between CNV burden and cognitive ability. Figure created with BioRender. CNVs: Copy Number Variants; AHBA: Allen Human Brain Atlas.

To understand the relationship between where genes are expressed in cortical networks and their impact on cognitive ability, we partitioned the coding genome into 180 gene-sets preferentially expressed in 180 regions of the human cerebral cortex based on the Glasser atlas^20^. To do so, we used normalized spatial profiles of gene expression from the Allen Human Brain Atlas^21^. A total of 13,678 genes with detectable expression in the cerebral cortex were assigned to cortical regions if their normalized value was higher than 0.5 z-score. As a result, we obtained 180 gene sets corresponding to 180 cortical regions (Fig. 1).

We then identified all CNVs > 50 kilobases in 258,513 individuals from 6 unselected populations with assessments of general cognitive ability (**Table ST1**). 8% of individuals carried at least one deletion/duplication fully encompassing one or more genes assigned to cortical regions (**Fig. S1c**). Among the 13,678 genes, 4,405 (32.2%) were deleted and 8,601 (62.9%) duplicated at least once (**Fig. S1a, Table ST4**). Given that deletion/duplication may occur multiple times, we observed a total of 28,108 and 77,695 deleted and duplicated genes, respectively (**Fig. S1, Table ST4**).

**Figure 2:**
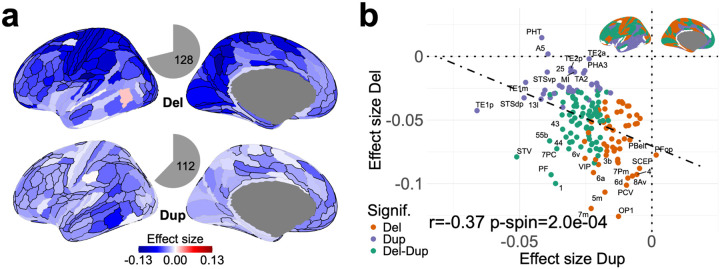
Cortical maps of deletion and duplication effect sizes on cognitive ability. **a)** Maps of deletion and duplication effect sizes in 180 cortical regions. We performed CC-GSBA for each cortical region, running 180 separate linear models. The color-coded effect sizes represent the average effect of genes with high expression in each region on Z-scored cognitive ability, mapped onto the cortex for both deletions and duplications. Regions with significant effects are outlined in black (p<0.05, Bonferroni corrected). The gray pie charts show the proportion of regions with significant effects; these include 128 regions for deletions and 112 regions for duplications. **b)** Negative correlation between the effect sizes of deletions and duplications across the cortex (spinpermutation p-value =2e-4). Each point is the mean effect size in the cortical regions. X-axis: duplication; Y-axis: deletion effect sizes, respectively. Only deletion or duplication significant effects are plotted, and corresponding significant regions are shown as a brain-plot in the top right corner. Del: deletion; Dup: duplication; Signif.: significance; r: Pearson correlation; p: spin permutation p-value.

We performed a cerebral-cortex gene-set burden analysis (CC-GSBA) by i) aggregating all CNVs affecting gene-sets assigned to cortical regions and ii) conducting 180 linear models to estimate the burden effect on cognitive ability of gene-sets assigned to each of the 180 cortical regions for deletions and duplications separately (Fig. 1). Most cortical regions showed (Bonferroni) significant negative associations (**Table ST3**) with cognitive ability for deletions (n=128) and duplications (n=112, Fig. 2). For deletions and duplications, the largest effect sizes were observed in sensorimotor (Cohen’s d = −0.12) and association (Cohen’s d = −0.06) regions, respectively (**Tables ST5, ST8**). Regions with the largest significant effects in deletions were those with the smallest (non-significant) effects in duplications and vice versa (Fig. 2). The group of regions (41%) significantly associated with both deletions and duplications included those clustered around the dorsolateral prefrontal cortex (DLPFC). These patterns of effect sizes across the cerebral cortex were robust and highly correlated between discovery, replication, and full datasets, as well as between the UK Biobank and the other unselected populations (**Fig. S2, Table ST9**).

## Deletions and duplications show mirror effects on cognitive ability across the cortex

It has been challenging to identify genes and biological processes with preferential sensitivity to either deletions or duplications. We therefore systematically compared the effect sizes of both CNV types across all cortical regions. We observed a negative spatial correlation between effect sizes of deletions and duplications (r = −0.37, spin permutation p-value=2E-4, Fig. 2b). Sensitivity analysis showed that these negative correlations (**Table ST6**) could also be observed using two independent gene expression resources (Human Protein Atlas (HPA)^14,15^ and BrainSpan^13^, **Fig. S3**), alternative methods for defining cortical gene sets^22^ (**Fig. S4**) or across discovery and replication data subsets (**Fig. S2**).

## Gene dosage effects on cognitive ability follow a sensorimotor-association cortical gradient

**Figure 3:**
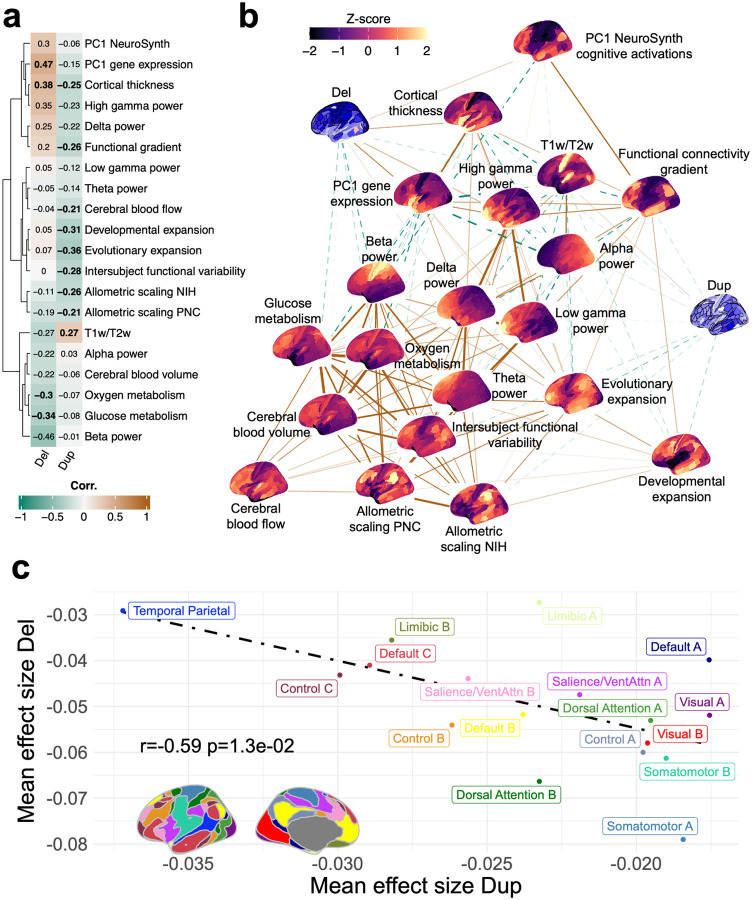
Deletion and duplication effect size maps align with sensorimotor-association cortical organizationhierarchy. **a)** Decoding the deletion and duplication effect size maps with 20 distinct neural maps^5,23^. The heatmap shows the spatial correlation between CNV effect size maps and 20 neuro-maps. Bold indicates significant spatial correlations (FDR-adjusted spin permutation p-value < 0.05). These normative cortical maps include two microstructural (cortical thickness, T1w/T2w), four metabolic (cerebral blood flow, cerebral blood volume, glucose metabolism, oxygen metabolism), three functional (functional gradient, PC1 NeuroSynth, intersubject functional variability), four expansion (evolutionary and developmental expansion, and allometric scaling NIH/PNC), six electrophysiological (alpha, beta, delta, low gamma, high gamma, theta), and one bulk transcriptomics (PC1 gene expression). The maps were obtained from the neuromaps toolbox^23^ and transformed to the Glasser parcellation. **b)** Spring embedding representation of the correlation matrix. The deletion and duplication maps, and the 20 neuro-maps, are shown as nodes. Positive and negative correlations are represented as solid and dotted lines. The width of the lines corresponds to the strength of the correlation. **c)** Resting state functional networks based decoding of the gene dosage effects across Yeo brain networks^24,68^. We mapped 180 cortical regions to the 17 Yeo brain networks to characterize the spatial patterns of effect sizes. This involved computing the mean effect size for each network, represented by a single point on the scatter plot. The dashed line shows the negative correlation between the mean effect sizes of deletions and duplications. The brain map in the bottom left outlines the 17 brain networks. We used the open-source BrainStat toolbox^24,68^ for resting state contextualization. Del: deletion; Dup: duplication; Signif.: significance; r: Pearson correlation; p: spin-permutation p-value.

To provide a functional interpretation of these gene dosage effect size maps, we annotated them using 20 reference neuromaps^23^. Overall, deletion and duplication effect size maps were aligned with the sensorimotor - association cortical gradients (Fig. 3a). Specifically, they showed positive and negative correlations, respectively, with multiple hierarchies of the cerebral cortex, including anatomical, cytoarchitecture, gene expression, functional connectivity, and electrophysiology gradients (Fig. 3a,b, **Table ST7**). A group of neuromaps were deletion or duplication specific: maps characterising evolutionary expansion of the cortex were associated with duplication, and conversely, maps characterizing metabolic processes were associated with deletions.

We further explored the functional interpretation of these maps by computing effect sizes across functional connectivity networks^24^. Again, the somatomotor and visual networks showed the largest effect sizes for deletions. In contrast, networks from the other end of the functional gradient (temporal-parietal and default mode networks) demonstrated the largest effects for duplications (Fig. 3c).

## Gene dosage effects on cognitive ability remain similar across levels of regional specificity

We reasoned that genes with the most specific regional expression were those driving the spatial patterns of gene dosage effect sizes across the cortical gradient. We therefore compared results from cortical burden tests at 4 levels of increasing specificity of regional expression. To do so, we thresholded normalized regional expression profiles at 5 levels of specificity: 0.2, 0.5, 0.8, and 1.0 Z-scores (thus creating smaller gene sets with higher regional specificity). Overall, the mirror effects between deletion and duplication effect size were strengthened (correlation increasing from −0.25 to −0.41, spin permutation p-value <0.05, **Table ST6**) with increasing regional specificity of gene expression. Deletions demonstrated a 1.4-fold increase (Welch test FDR p-value=2e-2) in effect size for genes with the higher sensorimotor specificity (**Table ST10**). A related analysis also demonstrated that genes with elevated brain expression were those driving the spatial distribution of effect sizes along the cortical gradient, compared to non-brain-elevated and ubiquitously expressed genes (**Fig. S8**). Multiple sensitivity analyses showed these spatial patterns were stable (**Tables ST9**) across i) data subsets (**Fig. S2**), ii) demographic parameters (sex, age, ancestry, **Fig. S6**), iii) different levels of tissue specificity (**Fig. S8a**), and iv) quality metric of gene expression data (stability score, **Table ST9**).

## Cortical patterns of gene dosage effect sizes are unrelated to genetic constraint, cell types, and developmental periods

**Figure 5:**
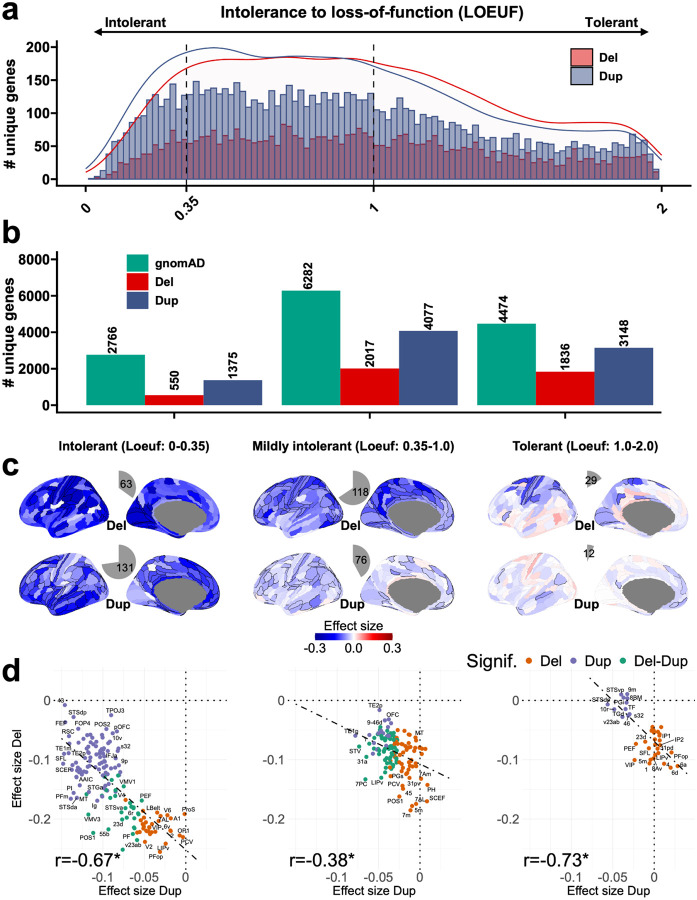
Genetic constraint and gene dosage cortical maps. **a)** Histogram showing the distribution of the number of genes deleted or duplicated at least once in our dataset, with respect to their genetic constraint (intolerance to loss of function; LOEUF^25^ values). Deletions and duplications are in red and blue. X-axis: 100 bins of LOEUF values. Density curves are added to compare the densities between deletions and duplications. **b)** Genes stratified into three LOEUF categories: i) highly intolerant (Loeuf1: 0–0.35); ii) intolerant (Loeuf2: 0.35–1.0); and iii) tolerant (Loeuf3: 1.0–2.0). Barplot showing the number (#) of unique genes observed in deletions and duplications in our data, for the three LOEUF categories. c) Cognitive ability effect size maps of deletions and duplications for the three LOEUF categories. Regions with significant effect sizes have black edges (Bonferroni p-value < 0.05). Grey edges are not significant. Pie charts show the number and proportion of significant cortical regions. The color scale shows the mean effect size of deletion or duplication on cognitive ability (Z-score) in that cortical region of interest. d) Negative correlation between the effect sizes of deletions and duplications across the cortex (*: FDR adjusted spin permutation p-value < 0.05. Each point is the mean effect size in the cortical regions. DEL: deletions; DUP: duplications; ES: effect size; r: Pearson correlation; Sig: significant.

We asked if the spatial distribution of gene dosage effects on cognitive ability was explained by genetic constraint measured by LOEUF^25^ (loss-of-function observed/expected upper bound fraction (LOEUF) score). This was not the case. We first show that genetic constraint does not correlate with the cortical gradient (**Fig. S7**). In other words, genes with a spatial pattern of expression following the gradient are not under higher constraint. (Fig. 5a–b). We then stratified genes into 3 groups: intolerant (LOEUF<0.35), mildly intolerant (0.35<LOEUF<1.0), and tolerant (LOEUF>1.0). As expected, increasing intolerance to loss of function was associated with increasing effect sizes on cognitive ability (Fig. 5–d, **Table ST5**). However, the spatial patterns of effect sizes remained similar and significantly anticorrelated between deletions and duplications for the three groups (−0.38 < r < −0.73, spin permutation p-value<1.3E-3, Fig. 5–d, **Table ST6**). These findings were unchanged using alternative dosage sensitivity scores^26^ (probability of haploinsufficiency (pHS), probability of triplosensitivity (pTS)) and constraint metrics (Missense Z-score^25^, **Fig. S9, Table ST6**).

We investigated more factors that could explain the gene-dosage cortical patterns described above. First, since bulk tissue expression data combine expression of underlying cell-constituents, we stratified genes based on 6 adult cell-types^14,15^, including Astrocytes, Oligodendrocyte precursor cells (OPCs), Oligodendrocytes, Microglial, Excitatory neurons, and Inhibitory neurons (Methods). Second, the temporal sequence of cortical maturation tends to progress along the sensorimotor-association cortical gradient^5,27^. We therefore stratified genes based on their temporal expression trajectories^28^ (prenatal, postnatal, and non-transitional). Overall, results show that the spatial patterns of effect sizes on cognitive ability remained similar and anticorrelated between deletions and duplications across all stratifications (**Fig. S8, Tables ST6, ST9**). This demonstrates that effects of gene dosage on cognitive ability are distributed according to large-scale brain networks, and these effects encompass and override the gene functions at the microscopic-cellular-level. Sensitivity analysis showed that molecular and sub-cellular biological functions (as detailed by GO-terms^29^) did not explain the pattern of CNV effects across the cerebral cortex (Methods, **Fig. S10, Tables S11, S12**).

## Discussion

This cortical region genetic burden analysis demonstrates that the effect size on cognitive ability of deleted and duplicated genes is strongly linked to their spatial expression across the cerebral cortex. This work identified a new gene dosage cortical axis. Genes with preferential expression in sensorimotor regions demonstrated the largest effect on cognition when deleted. At the opposing end of the cortical gradient, genes with preferential expression in multimodal association regions had the most significant impact on cognition when duplicated. These two opposing gene dosage cortical patterns could not be explained by particular cell types, developmental epochs, or genetic constraints.

Previous studies have shown that both deletions and duplications are associated with a decrease in cognitive ability^9,30,31^ (inverted U-shape effects, **Table ST2**). We did observe this inverted-U shape for a set of cortical regions (41%) clustered around the dorsolateral prefrontal cortex. However, half (51%) of the cortical regions were either sensitive to duplication or deletions^2,32^, with the largest effects on cognitive ability for deletions being observed in regions that showed smaller or near-zero effects for duplications, and vice versa. This mirror pattern suggests that opposing gene dosage alterations can lead to a decrease in cognitive ability through distinct cortical mechanisms.

Results demonstrate that a single cortical region, such as a motor region, can have either the largest or smallest effect on cognitive ability, depending on the class of genetic variant (deletion vs. duplication) disrupting genes preferentially expressed in that region. In studies where multiple classes of genomic variants (leading to different molecular effects) are aggregated at the gene level (i.e., gene-level burden or MAGMA), such effect size patterns would not be observed. This may also explain why brain-behaviour neuroimaging association studies have produced small effect sizes. In other words, if heterogeneous molecular mechanisms (with different cortical patterns) are driving inter-individual changes in cognitive ability, BWAS^33^ would therefore only capture the blurred average effect of these factors.

Our study introduces a new method to estimate aggregate genetic effects on a brain-wide scale, which we term cortical Gene Set Burden Analysis (CC-GSBA). This approach serves as an indirect method to interrogate the association between a trait and molecular changes in neuroanatomical regions. Our findings show that the effect sizes of deletions and duplications follow a sensorimotor-association axis. Deletion effects are largest in sensory motor regions that are among the first to develop during the late-fetal transition period^5^. This period is also enriched in many genes associated with psychiatric disorders, such as autism and schizophrenia^34–37^. In contrast, the largest effects of duplications involved genes with preferential expression in association regions characterized by protracted development from late childhood to adulthood^5^.

Our findings highlight primary sensorimotor regions as key contributors to general cognitive ability. We show that in the context of clear haploinsufficiency, these regions show the largest effects on cognitive ability. This aligns with recent research suggesting that the motor network plays a role not only in movement but also in cognitive functions like attention, working memory, and decision-making^38,39^. The observation of overconnectivity in somatomotor networks across psychiatric conditions^40^, cognitive ability, and CNVs is consistent with the fact that many rare loss-of-function variants associated with psychiatric risk are also linked to delayed gross motor milestones^41,42^ and developmental coordination disorders. The companion PGC-CNV paper^2^, which analyzes the risk conferred by CNVs for multiple psychiatric conditions, using the same Gene Set Burden Analysis (CC-GSBA) pipeline, also demonstrates the largest effects for deleted genes expressed in sensory motor regions for Schizophrenia, Attention Deficit Hyperactivity Disorder (ADHD), Major Depressive Disorder (MDD), and Autism Spectrum Disorder (ASD).

While studies have mapped common variants to genes preferentially expressed in the cerebral cortex^43^ using GWAS summary statistics, as well as run case-control transcriptomic differences^44^, they have primarily focused on the Dorsolateral prefrontal cortex. Our results show that this association is underpinned by complex spatial patterns across the cortex tied to two distinct classes of variants: deletions and duplications. We observed up to a 5-fold difference in effect sizes depending on where the genes are expressed across the cerebral cortex. This underscores the importance of variant-class consideration as well as moving beyond a DLPFC-centric view of transcriptomic studies to investigate the entire cerebral cortex and aligns with a recent transcriptomic study for ASD, albeit across 11 cortical regions^45^.

Finally, we show that the spatial patterns of gene dosage effects on cognitive ability cannot be explained by known contributors such as intolerance to loss of function. We demonstrate that constraint and cortical regional effects on cognition are orthogonal. Similarly, the mirror effects across the sensorimotor-association axis were not limited to brain-specific genes, suggesting that this “spatial organizing principle” regroups genes with multiple functions across the entire body. Stratification by cell type also showed the mirror effect across six major cell types. Moreover, the companion PGC-CNV paper^2^ demonstrated that cortical patterns of effect sizes obtained for multiple psychiatric disorders (including Schizophrenia, major depressive disorder, bipolar disorder, and ADHD) correlated with the sensorimotor-association axis.

Several limitations in our study are worth noting. The dataset’s large proportion of older adults may affect the interpretation of the results, making it difficult to distinguish between developmental and age-related effects and cognitive decline. The use of a spatial gene expression resource like the AHBA, which has sparse cortical sampling, required spatial interpolation, although each gene was assigned to several contiguous brain regions. However, the patterns of effect sizes were replicated using 2 independent RNA-seq resources covering the cerebral cortex. We also acknowledge that while our findings were robust across data subsets and were replicated in independent datasets, the smaller sample size for duplications, in particular, affects the reliability of some estimates.

Overall, this framework highlights the value of mapping genetic association results to the cerebral cortex using RNA-seq resources and concepts from structural, functional, and electrophysiological neuroimaging. Our gene dosage cortical patterns could not be explained by particular cell types, developmental epochs, or genetic constraints, highlighting the fact that the macroscopic network organization of the cerebral cortex is key to understanding the effects of gene dosage on cognitive traits.

## Methods

### Ethics

The study protocols were approved by the institutional review board for each of the cohorts involved with recruitment. Informed consent and permission to share the data were obtained from all individuals, in compliance with the guidelines specified by the institutional review boards of the recruiting centres. This study was approved by the ethics board of CHU Sainte Justine Research Center, Montreal, Canada.

### Participants and phenotype

The UK Biobank^46^ recruited ~500 000 individuals aged 40–69 years (54% female) between 2006 and 2010. A previous article by Kendall et al tested the impact of recurrent CNVs on many phenotypic and cognitive measures^47^. All procedures contributing to this work comply with the ethical standards of the relevant national and institutional committees on human experimentation and with the Helsinki Declaration of 1975, as revised in 2008. In this study, we used cognitive ability data from 258292 general population participants, described in previous publications from our lab^9,48^. Briefly, after phenotypic and genotypic quality control, we included 238176 participants from UK Biobank with fluid intelligence scores (February 2022 release), and 20116 participants from five general population cohorts (CARTaGENE (CaG)^49^, Generation Scotland (G-Scot)^50^, IMAGEN^51^, Lothian Birth Cohort (LBC)^52^, and Saguenay Youth Study (SYS)^53^) previously pooled and studied^9,48^ (**Table ST1**).

In the UKBB, fluid intelligence scores (FI scores) were assessed both in person (Data field 20016) and online (Data field 20191). The FI score is derived from 13 questionnaire items, assessing the capacity for logic and reasoning abilities, independent of acquired knowledge^47^. Each assessment consisted of participants completing as many questions as possible within an allotted time of 2 minutes. The obtained FI scores were transformed into a Z score using the mean and standard deviations for the subgroup assessed in person (mean=6.07, SD=2.15), and the subgroup assessed online (mean=6.61, SD=1.98).

We also computed the g-factor for the 5 cohorts and subset of UK Biobank participants, described in detail in previous work^48^. The g-factor is an indirect measure of general intelligence obtained by performing Principal Component Analysis (PCA) on a set of standardized cognitive measures and extracting the first unrotated principal component. Due to differences in the specific cognitive measures across the various tests (e.g., in-person vs. online in UKBB, or differences across cohorts), the g-factor was calculated and subsequently normalized to a Z-score separately within each test group and cohort using the mean and standard deviation computed from all available individuals in that group.

### Sample Processing and Genotyping

The DNA was extracted from blood. All details of the genotyping processes used are on the UK Biobank website. The samples were genotyped at the Affymetrix Research Laboratory, Santa Clara, CA, on 96-well plates. Samples were genotyped at Affymetrix Research Services Laboratory (Santa Clara, California, USA). Samples were genotyped on two very similar arrays with ~95% probe overlap between them. The first array did on ~50,000 on the UK BiLEVE Array (includes over 820,000 probes) and ~450,000 on the UK Biobank Axiom Array (includes over 807,000 probes). Quality control filters included genotypic call rate <0.95, |waviness factor| of <0.05, log R ratio s.d. of >0.35, and BAF s.d. of >0.08. From 488,377 people with genotypic data, 28,522 were excluded for failing these quality control filters, and if they were carrying a large CNV (≥ 10Mb or chromosome anomaly). We used only biallelic and common markers for both arrays for our CNV calls (~750,000 markers).

### CNV calling and quality controls

For all selected SNP array data, we called CNVs with PennCNV^54^ and QuantiSNP^55^ using previously published methods^9,10^ (https://martineaujeanlouis.github.io/MIND-GENESPARALLELCNV/#/). CNVs were called using both algorithms with the following parameters: number of consecutive probes for CNV detection ≥3, CNV size ≥1Kb, and confidence scores ≥15. CNVs detected by both algorithms were combined (CNVision^56^) to minimize the number of potential false discoveries. After this merging step, the CNV inheritance analysis algorithm (in-house algorithm), was applied to concatenate adjacent CNVs (same type) into one, according to the following criteria: a) gap between CNVs ≤150 kb; b) size of the CNVs ≥ 1000 bp; and c) number of probes ≥ 3. CNV filtering steps were previously published. After filtering the arrays according to their quality, we applied filtering for CNVs. The CNVs with the following criteria were selected for analysis: confidence score ≥ 30 (with at least one of both detection algorithms), size ≥ 50 kb, and unambiguous type (deletions or duplications). As part of a stringent quality control, every recurrent CNV was manually visualized by at least one individual.

### Annotation of CNVs

We annotated the CNVs using Gencode V19 annotation (the reference release for hg19 Human genome release) with ENSEMBL gene name (https://grch37.ensembl.org/index.html). Briefly, we used the bedtools suite (https://bedtools.readthedocs.io/en/latest/) to compare the different elements of the genes that overlap CNVs (UTRs, start and stop codons, exons, and introns). Thus, for each CNV, we obtained its proportion of overlap with each gene as well as with each gene component (UTR, Coding exons, start and stop codons, introns).

### Gene expression data

#### Allen Human Brain Atlas (AHBA) data:

The AHBA gene data matrix^8^ downloaded from (https://human.brain-map.org/static/download) was further processed using the abagen toolbox (version 0.1.3; https://github.com/rmarkello/abagen)^57^ based on recommendations in^58,59^. The following text was generated directly from abagen, describing the exact methodologies employed during preprocessing:

First, microarray probes were reannotated using data provided by^58^; probes not matched to a valid Entrez ID were discarded. Next, probes were filtered based on their expression intensity relative to background noise^21^, such that probes with intensity less than the background in >=50% of samples across donors were discarded. When multiple probes indexed the expression of the same gene, we selected and used the probe with the most consistent pattern of regional variation across donors (i.e., differential stability;^21^. The MNI coordinates of tissue samples were updated to those generated via non-linear registration using the Advanced Normalization Tools (ANTs; https://github.com/chrisfilo/alleninf). Inter-subject variation was addressed by normalizing tissue sample expression values across genes using a robust sigmoid function^60^. Normalized expression values were then rescaled to the unit interval. Finally, gene expression values were normalized across tissue samples using an identical procedure. Samples assigned to the same brain region were averaged separately for each donor and then across donors, yielding a regional expression matrix. After processing, a final 180 cortical regions × 15,633 gene matrix was used for subsequent analysis. We restricted to genes that were detected in the brain (list from the Human Protein Atlas^14^), leading to a final matrix of 180 cortical regions × 13678 genes.

#### BrainSpan-adult data (Independent replication resource):

processed using ABData package in R^61^. Briefly, RNA-seq data were grouped into 5 stages (fetal to adults)^13^, and the mean expression for 11 cortical structures was computed. We used the BrainSpan-adult (stage 5) data as an independent gene expression resource for replication.

#### Human Protein Atlas (HPA) data (Independent replication resource):

RNA-seq data from healthy adults processed using the pipeline proposed in the Human Protein Atlas^14^ (HPA v22). We obtained 73 regions × 16,000 gene matrix of normalized expression, and restricted it to the genes overlapping with our main analysis for subsequent analysis.

### Assigning genes to cortical brain regions

We assigned genes to cortical regions based on whether they were preferentially expressed (Z-score>0.5) in those regions (Fig. 1). Hereinafter referred to as preferentially-expressed-genes. Since genes with preferential expression were defined as Z-score>0.5, a gene was, on average, assigned to 52 out of 180 cortical regions.

### Assigning genes to developmental epochs

To investigate if our findings were dependent on the specific development stage (age) of gene-expression data, we stratified the adult gene-expression data into three non-overlapping gene sets with peak expression in different peak epochs. Briefly, genes were assigned to one of the three developmental epochs, based on BrainVar resource^28^: i) Epoch 1 ranges from 10 to 19 post-conception weeks (pcw); ii) Epoch 2: ranges between 19pcw and 6 postnatal months, and covers the late-fetal transition period, which has been hypothesised to be a critical window for many disorders^28^; and iii) Epoch 3: starts around 6 month and reaches all the way to adulthood.

### Assigning genes to temporal trajectories

Genes were also stratified based on their temporal trajectories, based on the BrainVar^28^ resource, into: i) falling genes; ii) non-transitional genes; and iii) rising genes.

### Brain and other tissue-specific gene sets

To investigate if our findings are driven by brain-elevated genes or can be reproduced irrespective of the tissue specificity of gene expression, we used the tissue-specific gene lists from the Human Protein Atlas (HPA, v22)^14^. The HPA performed a normalized transcriptome analysis of protein-coding genes across all major organs and tissue types in the human body, and compared mRNA levels. A gene is considered to have elevated expression based on either of the three conditions: i) Tissue enriched: At least four-fold higher mRNA level in a particular tissue compared to any other tissue.; ii) Group enriched: At least four-fold higher average mRNA level in a group of 2–5 tissues compared to any other tissue; and iii) Tissue enhanced: At least four-fold higher mRNA level in a particular tissue compared to the average level in all other tissues. We looked into genes that were brain-elevated (2685 genes), other-tissue elevated, and those that showed low-tissue specificity (https://www.proteinatlas.org/humanproteome/brain)^14^. Of note, we excluded the genes that were not detected in the brain as established by the HPA.

### Adult cell-type marker gene sets

Since bulk tissue expression data is an ensemble of underlying cell constituents, we looked into deconvolving our findings using cell-type marker gene sets defined in the HPA for major brain cell types^14^. The HPA used single-cell RNA sequencing (scRNAseq) data to cluster genes by their expression across major cell types. In this study, we focused on 6 major cell types in the brain: Astrocytes, Oligodendrocyte precursor cells (OPCs), Oligodendrocytes, Microglial, Excitatory neurons, and Inhibitory neurons. A genome-wide classification of all the protein-coding genes with regard to specificity was performed as follows: (i) cell type enriched genes with at least fourfold higher expression levels in one cell type as compared with any other analyzed cell type; (ii) group enriched genes with enriched expression in a small number of cell types (2 to 10); and (iii) cell type enhanced genes with only moderately elevated expression. We considered all genes that met any of these criteria for this study. The HPA confirmed the scRNA-seq profiles and cell type specificity using immunohistochemistry (IHC) on tissue microarrays.

### Genetic constraint-based gene sets

Previous studies have shown that measures of evolutionary/genetic constraint (such as LOEUF score^25^) explain a significant proportion of the effects of coding CNVs on cognitive and behavioral traits^9,16,17,62^. We then stratified genes into 3 non-overlapping groups based on genetic constraint: i) intolerant (LOEUF<0.35), ii) mildly intolerant (0.35<LOEUF<1.0), and iii) tolerant (LOEUF>1.0), and created the Glasser 180 cortical gene sets for these 3 categories. We applied this same strategy of stratifying into 3 non-overlapping groups for alternative dosage sensitivity scores^26^ (probability of haploinsufficiency (pHS or pHaplo), probability of triplosensitivity (pTS or pTriplo)) and constraint metrics (Missense Z-score^25^).

### Statistics

#### Spin permutation testing

Cortical maps of effect sizes were correlated by computing Pearson correlation. The statistical significance of such correlations was assessed using spin permutation^59,63^ testing, which accounts for spatial autocorrelation between brain regions. Since the neighboring cortical regions are expected to be similar, this approach avoids inflated significance values and generates plausible null brain maps for the null distribution. This may account for the overlap between the gene sets for neighboring cortical regions, as the cortical gene set overlap follows the spatial autocorrelation between brain regions.

#### Shrinkage for correlation of deletion/duplication effect size maps

We applied a shrinkage where cortical regions that were significant (Bonferroni multiple comparison correction) for either deletions or duplications were kept. This shrinkage ensured that cortical regions without significant estimates were not driving the spatial correlation.

#### Brain parcellations

We used the Glasser brain parcellation^20^ for our main analysis. Glasser parcellation was obtained using multi-modal magnetic resonance images from the Human Connectome Project (HCP)^64^, by delineating 180 areas per hemisphere bounded by sharp changes in cortical architecture, function, connectivity, and/or topography. In addition, we also investigated a functional cortical parcellation (Schaefer^65^) at different resolutions, as well as the Desikan^66^ parcellation.

#### Brain-maps using ggseg

All brain maps shown in this study were generated using the ggseg package^67^ in R.

#### Resting state contextualization

We used the BrainStat toolbox (https://brainstat.readthedocs.io/en/master/) for resting state contextualization. We mapped the effect sizes from the 180 cortical regions to 17 resting state brain networks from Yeo 17 parcellation^68^ by first mapping the 180 effect sizes to fsaverage5 vertices, and then averaging the effect sizes for 17 brain networks.

#### Established Cortical organization hierarchies

We used the previously published cortical organization hierarchies^5^, covering reference maps for molecular, microstructural, electrophysiological, developmental, and functional characterization of the human cortex. Deletion and duplication effect size maps of cognitive ability were annotated using 20 neural maps^5,23^, that included two microstructural (cortical thickness, T1w/T2w), four metabolic (cerebral blood flow, cerebral blood volume, glucose metabolism, oxygen metabolism), three functional (functional gradient, PC1 NeuroSynth, intersubject functional variability), four expansion (evolutionary and developmental expansion, and allometric scaling NIH/PNC), six band-specific electrophysiological signal power (alpha, beta, delta, low gamma, high gamma, theta), and one spatial transcriptomic map (PC1 gene expression). To compute a spatial correlation between deletion/duplication effect sizes maps of cognitive ability and the 20 neural maps, we obtained the 20 neural maps in fsavaerge5 space from the neuromaps package and mapped them to 180 cortical regions of Glasser parcellation by averaging values across all the vertices within a parcel. Finally, a spin permutation^23^ method was employed to assess the significance of the spatial correlation.

#### Sensorimotor and association cortical regions

To quantify the preference for the sensorimotor or association axes, we compared the effect sizes between the most sensorimotor or association regions using a previously established stratification approach^69^ (top 30% and bottom 30% regions of cortical gradient, **Fig. S5**). We used the anatomical organization hierarchy (T1w/T2w ratio) as the cortical gradient.

#### Enrichment in gene ontologies across cortical gene sets

The enrichment analyses encompassed a total of 1399 gene ontology (GO) terms^29^ from three categories: Gene Ontology biological process (GO-BP) with 1057 terms, Gene Ontology cellular component (GO-CC) with 177 terms, and Gene Ontology molecular function (GO-MF) with 165 terms. We used GO-term gene-sets from the Molecular Signatures Database^29^ (MSigDB, the msigdbr package in R, version 7.5.1), with gene-set size >50 and <700. The GO-term analyses encompassed three main steps. Firstly, GO enrichments were computed for each of the 180 brain regions and their corresponding gene sets using the ranked gene set enrichment method (the fgsea^70^ package in R). Secondly, enrichment scores were projected onto cortical maps to generate spatial profiles for each GO-term. Finally, spatial correlation analyses were conducted between the GO-term enrichment maps and the deletion and duplication effect size maps of cognitive ability. To assess the relationship between GO-term enrichment maps and the deletion effect size map, a spin permutation method was employed, controlling for false discovery rate (FDR, q<0.05). We restricted the spatial correlation analyses to those GO-term enrichment maps with >5 cortical regions showing significant enrichments (FDR q<0.05, across 1399 GO-terms × 180 cortical regions). The polarity of the deletion and duplication effect size maps was reversed before computing spatial correlation, so that the largest effect sizes are positive and correlated with positive enrichment scores.

## MATERIALS & CORRESPONDENCE

Requests for further information and resources should be directed to and will be fulfilled by the lead PI, Sebastien Jacquemont (sebastien.jacquemont@umontreal.ca).

## SUPPLEMENTARY MATERIALS

Supplementary Figs. S1–S10, Supplementary Tables S1–S12.

This is a list of supplementary files associated with this preprint. Click to download.

- SupplementaryTables.xlsx
- SupplementaryFigs.pdf

## Figures and Tables

**Figure 4:**
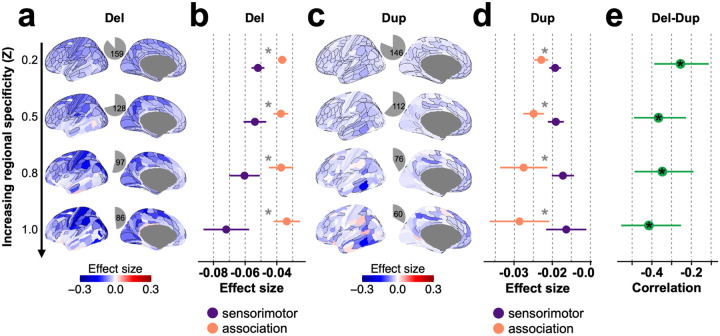
Gene dosage effects on cognitive ability across levels of regional specificity. **a,c)** Effect sizes maps of **a)** deletions and **c)** duplications on cognitive ability for four levels of regional specificity. Increasing the Z-score threshold (regional specificity parameter) leads to smaller regional gene-sets, which include genes showing higher regional specificity. Regions with significant effects are outlined in black (p<0.05, Bonferroni corrected for deletions and duplications for a given threshold). The gray pie charts show the proportion of regions with significant effects. **b,d)** Point-range plots comparing the mean effect sizes (and 95% confidence intervals of mean) of sensorimotor and association regions for **b)** deletions and **d)** duplications. *: represents a significant difference between the sensorimotor and association effect sizes (FDR q < 0.05). Cortical regions were ranked based on the established sensorimotor - association axis (Anatomical hierarchy, T1w/T2w)^5,23^. We examined the effect sizes of the top and bottom 30% of these regions^69^. The sensorimotor and association cortical regions are colored in purple and orange, respectively. **e)** Negative correlation between the effect sizes of deletions and duplications across the cortex. Each point is the Pearson correlation (and 95% confidence interval). Del: deletion; Dup: duplication; Signif.: significance;

## Data Availability

UK Biobank data was downloaded under the application 40980, and may be accessed via their standard data access procedure (see http://www.ukbiobank.ac.uk/register-apply). UK Biobank CNVs were called using the pipeline developed in the Jacquemont Lab, as described at https://github.com/labjacquemont/MIND-GENESPARALLELCNV. The final CNV calls are available for download from the UK Biobank returned datasets (Return ID: 3104, https://biobank.ndph.ox.ac.uk/ukb/dset.cgi?id=3104). References to the processing pipeline and R package versions used for analysis are listed in the methods.

## CODE AVAILABILITY

The code for generating all the Figures reported in the main analysis and supplement material can be found at the following GitHub link: https://github.com/kkumar-iitkgp/Mirror_effect_del_dup_cortex. Additional data (gene-sets) and code are archived on Zenodo: https://doi.org/10.5281/zenodo.17593039
